# Prevalence of antibodies against *Toxoplasma gondii* in pets and their owners in Shandong province, Eastern China

**DOI:** 10.1186/s12879-018-3307-2

**Published:** 2018-08-29

**Authors:** Wei Cong, Hany M. Elsheikha, Na Zhou, Peng Peng, Si-Yuan Qin, Qing-Feng Meng, Ai-Dong Qian

**Affiliations:** 1 Marine College, Shandong University at Weihai, Weihai, Shandong Province 264209 People’s Republic of China; 2 Faculty of Medicine and Health Sciences, School of Veterinary Medicine and Science, University of Nottingham, Sutton Bonington Campus, Loughborough, LE12 5RD UK; 3 The Affiliated Hospital of Qingdao University, Qingdao, Shandong Province 266000 People’s Republic of China; 4 General Station for Surveillance of Wildlife Diseases & Wildlife Borne Diseases, State Forestry Administration (SFA), Shenyang, Liaoning province 110000 People’s Republic of China; 5 College of Animal Science and Technology, Jilin Agricultural University, Changchun, Jilin Province 130118 People’s Republic of China; 6 Jilin Inspection and Quarantine Technology Center, Changchun, Jilin Province 130118 People’s Republic of China

**Keywords:** *Toxoplasma gondii*, Seroprevalence, Pet owners, Zoonosis, Eastern China

## Abstract

**Background:**

Pet ownership in China has been steadily increasing over recent years. However, the risk of pet-associated zoonotic infection with the protozoan parasite *Toxoplasma gondii* remains poorly defined.

**Methods:**

In a cross-sectional survey, we have determined the seroprevalence of *T. gondii* infection in pet dogs and cats, and pet owners. Serum samples were collected from 360 pets and 460 corresponding pet owners between March 2016 to June 2017, from Shandong province, eastern China. Sera from the animals were tested for anti-*T. gondii* antibodies using an indirect haemagglutination assay (IHA) and from the pet owners using an enzyme-linked immunosorbent assay (ELISA).

**Results:**

Antibodies against *T. gondii* were detected in 67 of 360 (18.61%) pets. Seroprevalence of *T. gondii* in pet cats and dogs was 21.67% and 15.56%, respectively. IgG and IgM antibodies were detected in 79 (17.17%) and 4 (0.87%) of pet owners, respectively; with a total of 83 of 460 (18.04%) pet owners testing seropositive for *T. gondii.* Our seroprevalence data also suggest that cat owners in general and female pet owners in particular could face a higher risk of acquiring *T. gondii* infection.

**Conclusions:**

Significant levels of anti-*T. gondii* antibodies were detected in the pets and their owners in Shandong province, eastern China, indicating a potential zoonotic risk. Prophylactic measures should be implemented to reduce the risk of pet owner’s exposure to *T. gondii* infection.

## Background

Significant strides have been made in the prevention and treatment of parasitic diseases. However, many parasites remain a common cause of morbidity and mortality in pet animals and can also pose a threat to the environmental and public health [1]. One of these parasitic diseases is toxoplasmosis, an important zoonotic parasite disease caused by the opportunistic protozoan *Toxoplasma gondii*. This parasite can infect almost all domesticated and wild animals, and humans [2, 3]*.* Cats represent the only definitive host of *T. gondii,* with many animals, including dogs, serving as the intermediate host. Humans acquire *T. gondii* infection via ingestion of undercooked or raw meat containing *T. gondii* cysts, or through ingesting food or water contaminated with cat-derived *T. gondii* oocysts [3, 4].

Cats and dogs are the most popular pets, and play a significant role in people’s daily life [5] - bringing many benefits to humans’ physical and mental health [6]. In China, pet ownership is becoming more common [7]; with rapid economic and social development. This increase in pet ownership has been considered as an indicator of the improved socioeconomic status of the Chinese society [8]. The population of cats and dogs being kept as pets in China, are approaching 100 and 200 million, respectively [9]. In Beijing, the number of pet dogs increased from 100,000 to 1.5 million between 2001 and 2007. Dogs and cats are also used in rural areas of China to guard homes and limit the spread of rats.

Despite the increasing popularity of dogs and cats as pets, they can be a source of many zoonotic diseases to humans [10–13]; especially children, the elderly and immunocompromised individuals [14–16]. Both humans and pets (dogs and cats) are known to be susceptible to infection with the agent that causes toxoplasmosis. A recognition of the potential transmission of many parasitic zoonoses from pets to humans has led to a worldwide increase in the number of studies of *T. gondii* infection in pets [4, 17, 18] - including mainland China [19, 20]. However, nothing is known about the prevalence of *T. gondii* infection in pet owners in China.

The aim of the present study, was to determine the seroprevalence of *T. gondii* infection in pet dogs and cats, and their owners, in Shandong province, eastern China. The serological findings provide evidence that pet ownership can significantly increase the risk for pet-associated zoonotic *T. gondii* infection in humans.

## Methods

### Sample collection

The study was conducted from March 2016 to June 2017 and included 360 pets (180 dogs and 180 cats) and 460 pet owners (184 males and 176 females) from three regions in Shandong province (4°23′~ 38°24′ N; 114°48′~ 122°42′ E). Blood samples were collected from the medial saphenous vein of the pet cats and dogs by local veterinary practitioners, after a written informed agreement was obtained from the pet owners. Blood samples were also collected form the venous blood from the pet owenrs by a medical practitioner. All blood samples were left overnight at ambient temperature, followed by centrifugation at 1,500×*g* for 10 min to separate the serum from the blood clot. The sera of pets, and their owners, were collected in Eppendorf tubes and stored at − 20 °C, until serological analysis.

### Serological examination

Dog and cat sera were tested for anti-*T. gondii* antibodies using an indirect haemagglutination test (IHA). The test was performed according to the instructions provided in the kit, developed by Lanzhou Veterinary Research Institute, Chinese Academy of Agricultural Sciences, Lanzhou, Gansu Province, China. Reactions obtained at 1:64 or greater dilutions of serum were considered positive. Pet owner’s sera were analyzed for the presence of IgG and IgM antibodies against *T. gondii*, using the commercially-available ELISA kits, in accordance with the manufacturer’s instructions (Demeditec Diagnostics GmbH, Germany). Optical densities were determined using a wavelength of 450 nm. Values higher than the cut-off (10 IU/ml) were considered positive. Values within ±20% of the cut-off were considered to be equivocal, and these samples were retested. Positive and negative control sera were included on each microtiter plate. Serological testing was performed by two individuals, blinded to the experimental design to avoid bias.

### Statistical analysis

Statistical analysis was performed using SAS version 9.3. A Chi-square test was used to compare the percentages, with results considered to be statistically significant if *P* value < 0.05.

## Results

### Seroprevalence of T. gondii in the pet dogs and cats

The overall prevalence, and prevalence per variable, of anti-*T. gondii* antibodies are shown in Table 1. Serologically-positive sera were detected in 67 of the 360 examined pets (18.61%), with titers of 1:64 found in 24 pets, 1:128 in 16 pets, 1:256 in 12 pets, 1:518 in 11 pets and 1:1024 in 4 pets (Table 1). *T. gondii* seroprevalence in cats and dogs were 21.67% and 15.56%, respectively. *T. gondii* seroprevalence in pets from Weihai, Yantai and Rizhao were 19.17%, 19.17% and 17.50%, respectively. The greatest *T. gondii* seroprevalence was found in the 3-year old age pets (28.26%), followed by the ≤1-year old age pets (18.25%), and > 3-year old age pets (17.72%). The lowest seroprevalence was found in the 2-year old age pets (15.31%). Male pets (20.11%) had a higher *T. gondii* seroprevalence than female pets (17.05%); however the difference was not statistically significant (*P* > 0.05). Pets known to come from urban areas (18.62%) had a similar seroprevalence with those from rural areas (18.6%).

**Table 1:** Seroprevalence of *T. gondii* infection in pet dogs and cats in Eastern China, using an indirect haemagglutination test

Variable	Distribution of titers/No. of pets					Pos/*n* (seroprevalence %)^a^	*P*-value
	1:64	1:128	1:256	1:512	1:1024		
Pet species							
Cat	13	9	7	7	3	39/180 (21.67)	0.136
Dog	11	7	5	4	1	28/180 (15.56)	
Region							
Weihai	8	6	4	3	2	23/120 (19.17)	0.929
Qingdao	7	6	4	4	2	23/120 (19.17)	
Rizhao	9	4	4	4	0	21/120 (17.50)	
Age (year)							
≤ 1	7	7	6	3	2	25/137 (18.25)	0.310
2	8	2	3	2	0	15/98 (15.31)	
3	3	5	0	4	1	13/46 (28.26)	
> 3	6	2	3	2	1	14/79 (17.72)	
Gender							
Male	12	12	6	4	3	37/184 (20.11)	0.455
Female	12	4	6	7	1	30/176 (17.05)	
Residence area							
Urban	8	6	7	4	2	27/145 (18.62)	0.997
Rural	16	10	5	7	2	40/215 (18.60)	
Total	24	16	12	11	4	67/360 (18.61)	

^a^Pos, number of positive samples; *n*, total number of samples

### Prevalence of and demographic variables associated with T. gondii in pet owners

ELISA was used to detect *T. gondii* antibodies in pet owners, and 83 of 460 pet owners (18.04%), were seropositive for *T. gondii. T. gondii* IgG and IgM antibodies were found in 79 (17.17%) and 4 (0.87%) of the pet owners, respectively. As shown in Table 2, the highest *T. gondii* seroprevalence was found in the 21- to 30-year old age group (20.62%), followed by the 41- to 50-year old age group (19.78%) and > 50-year old age group (18.31%). The lowest seroprevalence was found in the ≤20-year-old age group (15.49%). *T. gondii* seroprevalence in pet owners from Weihai, Yantai and Rizhao were 16.67%, 21.62% and 15.97%, respectively. Cat owners (22.32%) had a significantly higher seroprevalence than dog owners (13.66%) (*P* = 0.016). Moreover, female owners (24.39%) had a significantly higher seroprevalence than male owners (10.75%) (*P* < 0.001). Pet owners living in rural areas (19.78%) had a higher seroprevalence than those living in urban areas (15.51%), however the difference was not statistically significant (*P* > 0.05). Pet owners who used their bare hands to dispose of pet feces (18.97%), had a higher seroprevalence when compared with those who used gloves to discard pet feces (12.86), however the difference was not significant (*P* > 0.05).

**Table 2:** Seroprevalence of *T. gondii* infection in pet owners in Eastern China, revealed by ELISA

Variable	Pos/*n* (seroprevalence %)^a^	*P*-value
Age (year)		
≤ 20	11/71 (15.49)	0.870
21–30	20/97 (20.62)	
31–40	21/130 (16.15)	
41–50	18/91 (19.78)	
> 50	13/71 (18.31)	
Region		
Weihai	28/168 (16.67)	0.384
Qingdao	32/148 (21.62)	
Rizhao	23/144 (15.97)	
Pet species		
Cat	52/233 (22.32)	0.016
Dog	31/227 (13.66)	
Gender		
Female	60/246 (24.39)	< 0.001
Male	23/214 (10.75)	
Residence area		
Urban	29/187 (15.51)	0.242
Rural	54/273 (19.78)	
Disposal of pet feces		
Using gloves	9/70 (12.86)	0.220
Using hands	74/390 (18.97)	
Total	83/460 (18.04)	

^a^Pos, number of positive samples; *n*, total number of samples

## Discussion

In the present study, the serological evidence for the presence of *T. gondii* infection in pet dogs and cats, and their owners in Shandong province, eastern China is reported. We demonstrated that *T. gondii* seroprevalence in pets and pet owners was 18.61% and 18.04%, respectively. Seroprevalence of *T. gondii* in cats was 21.67%, being slightly lower than the 24.5% seroprevalence detected in cats in mainland China, from 1991 to 2015 [20]. Seroprevalence *T. gondii* in dogs was 15.56% - higher than the 11.1% seroprevalence previously reported in pet dogs in China [19].

The association between cat ownership and increased seropositivity of *T. gondii* is to be anticipated, because cats - serving as the definitive host of this parasite - play a key role in the transmission of *T. gondii* to humans [21]. In the studied regions, cats are used mainly to control rats and mice, or as a pet. Cats become infected with *T. gondii* when they prey on infected rodents or birds. This is followed by excretion of millions of environmentally-resistant oocysts in cat feces, causing health risks to animals and humans [3, 22]. The presence of cats at home increases the risk of exposure to *T. gondii*, as reported previously [23].

Dogs, on the other hand, are a dead-end intermediate host and do not produce *T. gondii* *T. gondii* oocysts; and might be considered less important to the zoonotic transmission of *T. gondii*. However, dogs can ingest oocysts from cat feces, by coprophagia, which pass intact [24] and even viable [25] through their gut into the feces. Some dogs have a tendency to roll in pungent substances, such as cat feces. This behaviour means that dogs can mechanically spread *T. gondii* oocysts, contaminating the environment [24] and infecting humans - accidentally through ingestion of oocysts from dog fur while petting them [26]. The ingestion of improperly-cooked infected meat, can be a source of transmission to people in areas where selling dog meat for human consumption is permitted [27].

Our study also found that female pet owners (24.39%, 60/246) had a significantly higher seroprevalence than male pet owners (10.75%, 23/214; *P* < 0.001) (Table 2). This may be due to various factors. In China, women are responsible for taking care of pet animals at home, handling raw meat and vegetables, and performing household cleaning tasks. These factors can increase their contact with cats and dogs, and their feces, thus increasing opportunities of acquiring the infection compared with men. With this in mind, women should follow basic hygienic measures to limit exposure to zoonotic agents; including wearing gloves and masks when handling cat feces, together with hygienic disposal of cat feces and frequent hand-washing - particularly before preparing food.

There is increasing evidence for the importance of waterborne transmission of *T. gondii* to humans, via the dissemination of oocysts through surface water [28]. Therefore, proper disposal of cat litter can reduce environmental contamination with oocysts excreted in cat feces [29]. Although pet owners who used their hands to dispose pet feces (18.97%) had a higher seroprevalence compared with those who used gloves (12.86%), the difference was not statistically significant (*P* > 0.05). This may be due to a large difference in the sample-size between people who disposed pet feces using gloves, and those using bare hands. Regardless, cat feces must be hygienically disposed of, in order to reduce the environmental contamination with *T. gondii* oocysts.

In China, approximately 1 in 5 pet cats is *T. gondii*-seropositive, and this may impose a significant risk as the number of owners, and their interactions with cats, is increasing [20]. Cats and dogs are known to harbor other protozoal species [17], helminthes [30], and many other types of pathogens, such as fungal, viral, and bacterial agents [31], and may constitute potential reservoirs to their owners. Therefore, the epidemiological and public health importance of zoonotic infections, in pet dogs and cats in China, should be investigated in future studies. In the interim, we advocate the implementation of better measures to improve personal hygiene; and to maintain good pet-keeping management practice, which will ultimately help in reducing the risk of *T. gondii* infection, and other zoonoses, in humans.

## Conclusions

The present results show that the seroprevalence of *T. gondii* infection in pets and their owners in Shandong province, eastern China, was high and poses significant health risks to the local community. Our study provides evidence, that an association exists between the ownership of pet cats or dogs, and an increased risk for *T. gondii* infections in pet owners. Therefore, it is imperative to implement control measures to reduce *T. gondii* prevalence in pets, and pet owners, in the studied regions and elsewhere in China. It remains to be determined, whether or not pets and their owners may have been exposed to the same environmental source of *T. gondii* infection; and the directionality of transmission between pets and pet owners requires further investigation.

## Abbreviations

ELISA: Enzyme-linked Immunosorbent Assay
IHA: Indirect Haemagglutination Test
SAS: Statistical Analysis System
